# Supplementary material for: Therapeutic Textiles Functionalized with Keratin-Based Particles Encapsulating Terbinafine for the Treatment of Onychomycosis
Source: Int J Mol Sci. 2022 Nov 13;23(22):13999. doi: 10.3390/ijms232213999 (PMC9699589; doi:10.3390/ijms232213999)
Supplement: Supplementary file 1 [file ijms-23-13999-s001.zip › ijms-2013067-supplementary.pdf]

International Journal of
Molecular Sciences

MDPI

Supplementary Table S1 – Antimicrobial and antifungal activity of cotton fabrics functionalized with keratin-based particles (100% keratin-Terb; 80% keratin/20%keratin-PEG-Terb) against Trichophytan rubran, Candida parapsilosis, Escherichia coli, and Staphylococcus aureus.

Microorganism	Activity
**Trichophytan rubran**	+
**Candida parapsilosis**	-
**Escherichia coli**	-
**Staphylococcus aureus**	-
